# Dysfunction of CD8 + PD-1 + T cells in type 2 diabetes caused by the impairment of metabolism-immune axis

**DOI:** 10.1038/s41598-020-71946-3

**Published:** 2020-09-10

**Authors:** Ichiro Nojima, Shingo Eikawa, Nahoko Tomonobu, Yoshiko Hada, Nobuo Kajitani, Sanae Teshigawara, Satoshi Miyamoto, Atsuhito Tone, Haruhito A. Uchida, Atsuko Nakatsuka, Jun Eguchi, Kenichi Shikata, Heiichiro Udono, Jun Wada

**Affiliations:** 1 Department of Nephrology, Rheumatology, Endocrinology and Metabolism, Okayama University Graduate School of Medicine, Dentistry and Pharmaceutical Sciences, 2-5-1 Shikata-cho, Kitaku, Okayama City, 700-8558 Japan; 2 Department of Hematology/Oncology, Hess Cancer Institute, Icahn School of Medicine At Mount Sinai, New York, USA; 3 Department of Cell Biology, Okayama University Graduate School of Medicine, Dentistry and Pharmaceutical Sciences, Okayama, Japan; 4 Department of Internal Medicine, Diabetes Center, Okayama City Hospital, Okayama, Japan; 5 Diabetes Center, Okayama Saiseikai General Hospital, Okayama, Japan; 6 Center for Innovative Clinical Medicine, Okayama University Hospital, Okayama, Japan; 7 Department of Chronic Kidney Disease and Cardiovascular Disease, Okayama University Graduate School of Medicine, Dentistry and Pharmaceutical Sciences, Okayama, Japan; 8 Department of Immunology, Okayama University Graduate School of Medicine, Dentistry and Pharmaceutical Sciences, Okayama, Japan

**Keywords:** Immunology, Cytokines, Tumour immunology, Endocrine system and metabolic diseases, Diabetes

## Abstract

The metabolic changes and dysfunction in CD8 + T cells may be involved in tumor progression and susceptibility to virus infection in type 2 diabetes (T2D). In C57BL/6JJcl mice fed with high fat-high sucrose chow (HFS), multifunctionality of CD8 + splenic and tumor-infiltrating lymphocytes (TILs) was impaired and associated with enhanced tumor growth, which were inhibited by metformin. In CD8 + splenic T cells from the HFS mice, glycolysis/basal respiration ratio was significantly reduced and reversed by metformin. In the patients with T2D (DM), multifunctionality of circulating CD8 + PD-1 + T cells stimulated with PMA/ionomycin as well as with HLA-A*24:02 CMV peptide was dampened, while metformin recovered multifunctionality. Both glycolysis and basal respiration were reduced in DM, and glycolysis was increased by metformin. The disturbance of the link between metabolism and immune function in CD8 + PD-1 + T cells in T2D was proved by recovery of antigen-specific and non-specific cytokine production via metformin-mediated increase in glycolytic activity.

## Introduction

In international diabetes federation (IDF) Diabetes Atlas, it was estimated that 451 million adults (age 18–99 years) are suffered from diabetes worldwide and they were expected to increase to 693 million by 2045^1^. In 2017, 5 million deaths were attributed to diabetes and global healthcare expenditure was estimated to be USD 850 billion^1^. Although diabetic micro- and macro-vascular complications are major causes of death in diabetes patients, it has been suggested that there is a tight association between diabetes and carcinogenesis such as pancreatic, hepatic, colorectal, breast, urinary tract and endometrial cancers^2^. Hazard ratios in the patients with diabetes at HbA1c levels ≥ 6.5% compared with individuals without known diabetes (HbA1c levels 5.0–5.4%) were 1.43 (1.14–1.80) in Japanese population^3^. Hyperinsulimemia, hyperglycemia, and obesity-induced chronic inflammation have been speculated to be possible biological basis for the link between diabetes and cancer^4^.

The cancer cells have been long known to undergo alterations in glucose and amino acid metabolism to respond to bioenergic needs for rapid proliferation, *i.e.* metabolic reprogramming. They shift their metabolism to a glycolytic mode even under aerobic conditions, known as Warburg effect^5^. Recently, the immune cells have been extensively investigated how they undergo metabolic reprogramming during the process of tumorigenesis. For example, memory T cells at quiescence state generate most of their energy by fatty acid oxidation (FAO) and oxidative phosphorylation (OXPHOS), while effector T cells upon activation mainly utilize the glycolysis and glutaminolysis to maintain rapid proliferation^6^. Under tumor microenvironment, lack of antigen recognition, impaired boosting of T cells, and chronic activation cause the failure of T cell to protect against cancer. Under tumor hypoxia and hyponutrition, most T cells are exhausted and T-cell exhaustion is characterized by increased inhibitory receptors such as programmed cell death protein 1 (PD-1), decreased effector cytokines and impaired cytotoxicity^7^. Vigorous glucose consumption by tumor cells metabolically compete with infiltrating T cells and hyponutrition in T cells reduced mammalian target of rapamycin (mTOR) activity, glycolysis and cytokine production such as interferon (IFN)-γ. The blocking antibodies against immune checkpoint molecules, CTLA-4, PD-1, and PD-L1, restore glucose in tumor cells and permit T cell glycolysis and IFN-γ production^8^.

The lines of evidence suggested that metabolic modulators are therapeutic candidates for the prevention and treatment for cancer and metformin is highlighted as one of the anti-aging and anti-cancer metabolic modulators^9^. Metformin was released back in 1959 and ranked as top-prescribed anti-diabetic agent since the United Kingdom Prospective Diabetes Study (UKPDS) reported that metformin-allocated patients demonstrated the 33% reduction of cardiac infarction and 27% reduction of death from any causes^10^. Furthermore, meta-analysis demonstrated that metformin users were significantly lower than those among non-metformin users: the pooled RRs (95% confidence interval) were 0.66 (0.49–0.88) for cancer mortality and 0.67 (0.53–0.85) for all-cancer incidence^11^. Metformin may exert anti-cancer effects reduced the insulin/IGF-1 receptor-mediated activation of phosphatidylinositol 3-kinase (PI3K) pathway through the activation of the AMP-activated protein kinase (AMPK)^12^; however, the precise mechanism is totally unexplored. Eikawa et al. reported that metformin increased the number of CD8(+) tumor-infiltrating lymphocytes (TILs) and protected them from apoptosis and dysfunction, and increased the production of IL-2, TNFα, and IFN-γ in mouse model^13^. Although the neutrophil dysfunction in the patients with diabetes, such as adhesive dysfunction^14^, impaired chemotaxis^15^, and reduced bactericidal capacity^16^ were reported, no data are available peripheral CD8 + T cells in the patients with type 2 diabetes (T2D). The metabolic changes and immune dysfunction in peripheral CD8 T cells may be involved in tumor progression and susceptibility to virus infection in diabetes^17,18^. Here, we investigated peripheral CD8 + T cells derived from the patients with T2D and their alterations by the treatment with metformin by evaluating the cytokine production and metabolic states by flux analyzer.

## Results

### Enhanced tumor growth and its suppression by metformin in mice with diabetes

**Figure 1:**
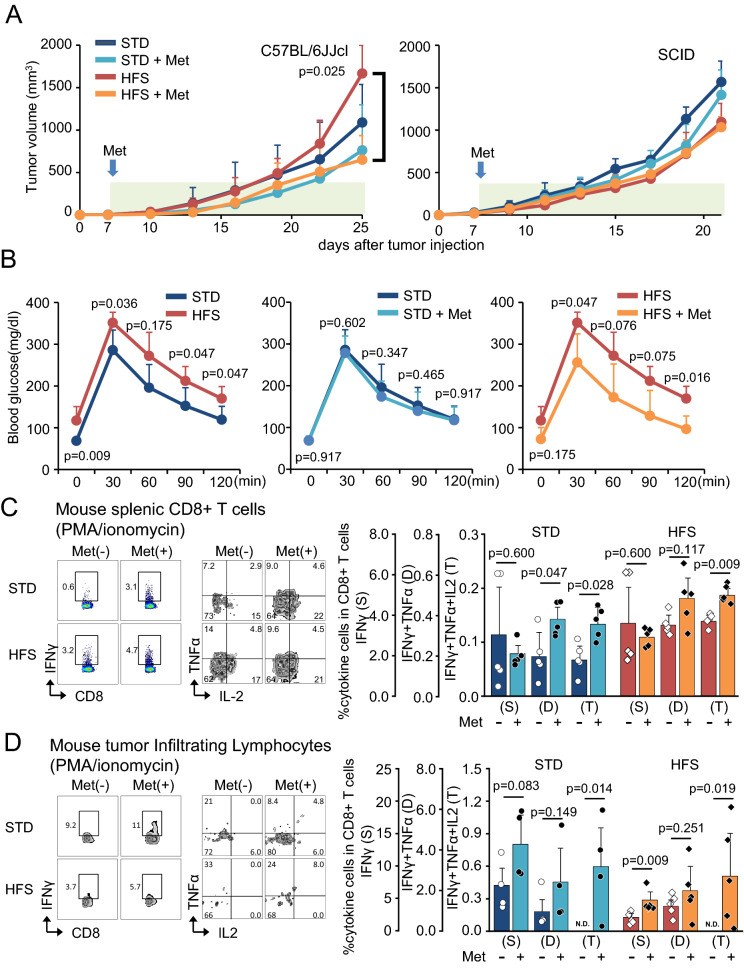
C57BL/6JJcl mice injected with B6 OVA-gene introduced B16 melanoma MO5 cells. (**A**) Time course of tumor volume in C57BL/6JJcl and B6.CB17-Prkdc^scid^/SzJ (SCID) mice. Standard chow (STD; n = 5), high fat-high sucrose chow (HFS; n = 5), STD and metformin (STD + Met; n = 5), and HFS and metformin (HFS + Met; n = 5). (**B**) Intraperitoneal glucose tolerance tests at 12 weeks after HFS feeding. STD (n = 5), HFS (n = 5), STD + Met (n = 5), and HFS + Met (n = 5). Area under the curve (AUC); STD, 364 (± 66.8) mg/dL/min; HFS, 490 (± 60.3) mg/dL/min; STD + Met, 343 (± 56.8) mg/dL/min; HFS + Met, 321 (± 110) mg/dL/min. STD versus HSF, p = 0.028; STD versus STD + Met, *p* = 0.754; HFS versus HFS + Met, *p* = 0.047. (**C**) Mouse splenic CD8 + T cells treated with PMA/ionomycin. The percentage of single (S) IFNγ, double (D) IFNγ + TNFα, and triple (T) IFNγ + TNFα + IL2 cytokine producing cells in CD8 + T cells. STD (n = 5), HFS (n = 5), STD + Met (n = 5), and HFS + Met (n = 5). (**D**) Mouse tumor infiltrating lymphocytes (CD8 + T cells) treated with PMA/ionomycin. The percentage of single IFNγ (S), double (D) IFNγ + TNFα, and triple (T) IFNγ + TNFα + IL2 cytokine producing CD8 + T cells. STD (n = 5), HFS (n = 5), STD + Met (n = 4), and HFS + Met (n = 5). (**A**–**D**, Mann–Whitney U test).

Since the incidence of malignancies increases in the patients with T2D and metformin reduces the rate of cancer development and mortality, we first investigated whether solid tumor growth is enhanced in diet-induced diabetes model of mice and whether metformin demonstrates anti-cancer effects. Four-week-old C57BL/6JJcl fed with standard chow diet (STD) or high fat-high sucrose diet (HFS) were intradermally injected with B6 OVA-gene introduced B16 melanoma MO5 cells. After seven days, they were divided into STD, STD + Met, HFS and HFS + Met groups. In C57BL/6JJcl mice, the tumor sizes tended to be increased in HFS compared to STD. Significant reduction of tumor size by metformin treatment was observed in HFS compared to HFS + Met (Fig. 1A, left panel). In SCID mice, such anti-tumor effects were completely abolished, suggesting the requirement of T and/or B cells in metformin-mediated tumor suppression (Fig. 1A, right panel). C57BL/6JJcl mice fed with STD and HFS demonstrated no differences in body weight and blood glucose levels 6 weeks after HSF feeding, while the significant body weight gain (online supplementary Figure S1A), insulin resistance (online supplementary Figure S1B) and glucose intolerance (Fig. 1B) developed at 12 weeks after HFS feeding. In this glucose tolerance test, blood glucose levels and area under the curve (AUC) were elevated in HFS compared with STD, while glucose tolerance was improved in HFS + Met (Fig. 1B).

### Metformin improved the multiple cytokine production from CD8 + cells in mice

In previous investigation, we reported that metformin improved multifunctionality of CD8 + tumor-infiltrating lymphocytes (TILs), *i.e.* simultaneous production of IL2, TNFα, and IFNγ^13^. We next investigated the multifunctionality of CD8 + splenocytes in C57BL/6JJcl mice fed with HFS and the effects of metformin on multifunctionality. The splenocytes were isolated from C57BL/6JJcl mice consisted of 4 groups, STD, STD + Met, HFS, and HFS + Met, stimulated with PMA/ionomycin for 6 h, and subjected cytokine assay. Under STD, metformin significantly increased the population of CD8 + splenocytes producing double (IFNγ + TNFα) and triple (IFNγ + TNFα + IL2) cytokines. Similarly, C57BL/6JJcl mice fed with HFS demonstrated the significantly increased number of CD8 + splenocytes producing IFNγ + TNFα + IL2 by the treatment of metformin (Fig. 1C). TILs were isolated from C57BL/6JJcl mice injected with MO5 cells and fed with STD, STD + Met, HFS, and HFS + Met. The population of CD8 + TILs producing IFNγ + TNFα + IL2 was absent in both STD and HFS groups, they appeared in STD + Met, and HFS + Met groups (Fig. 1D). The ratio of CD8 + TILs producing single cytokine (IFNγ) was also significantly increased in C57BL/6JJcl mice fed with and HFS (Fig. 1D). In the comparison between STD and HFS groups without the treatment of metformin, it is intriguing to note that CD8 + splenocytes producing IFNγ + TNFα + IL2 were significantly increased (online supplementary Figure S1C), while CD8 + TILs producing IFNγ were significantly reduced (online supplementary Figure S1D). It suggested that increased population of CD8 + splenocytes producing triple cytokines under HFS reflects systemic inflammation induced by diabetes and obesity, while multifunctionality of CD8 + TILs may be impaired by tumor environment induced by enhanced tumor growth under obesity and hyperinsulinemia.

### Impaired multifunctionality of peripheral CD8 + T cells in patients with T2D

**Figure 2:**
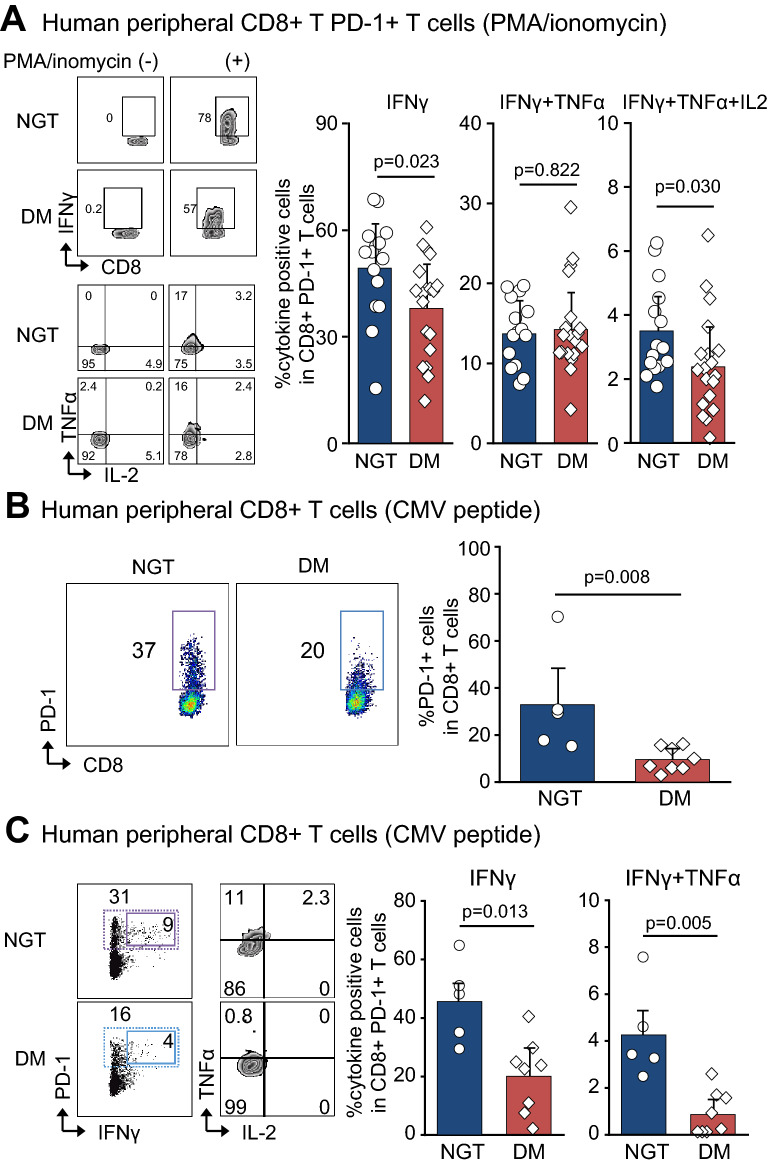
Human peripheral CD8 + T cells isolated from the subjects with normal glucose tolerance (NGT) and the patients with type 2 diabetes (DM). (**A**) Human peripheral CD8 + T cells stimulated with PMA/ionomycin. The percentage of single (S) IFNγ, double (D) IFNγ + TNFα, and triple (T) IFNγ + TNFα + IL2 cytokine producing cells in CD8 + T cells. NGT (n = 15) and DM (n = 19). (**B**) Human peripheral CD8 + T cells stimulated with HLA-A*24:02 CMV pp65 peptide. The percentage of PD-1 + cells in CD8 + T cells in NGT (n = 5) and DM (n = 8) is shown. (**C**) Human peripheral CD8 + T cells stimulated with HLA-A*24:02 CMV pp65 peptide. The percentage of IFNγ and IFNγ + TNFα producing cells in CD8 + PD-1 + T cells is shown. NGT (n = 5) and DM (n = 8). (**A**–**C**, Mann–Whitney U test).

In experiments in mice, metformin demonstrated anti-tumor effects by increasing the population of both CD8 + splenocytes and TILs producing multiple cytokine. We further investigated the multifunctionality of peripheral CD8 + T cells in the patients with T2D Peripheral CD8 + T cells derived from subjects with age- and gender-matched normal glucose tolerance (NGT, n = 15) and patients with T2D (DM, n = 19) (online supplementary Table S1) were stimulated by PMA/ionomycin (PMA/ION) for 6 h and subjected to cytokine assay. In gated population of CD8 + T cells, there were no differences in the population of CD8 + T cells producing IFNγ, IFNγ + TNFα and IFNγ + TNFα + IL2 (online supplementary Figure S2). However, in gated CD8 + PD1 + T cells, the populations producing IFNγ and IFNγ + TNFα + IL2 were significantly reduced in DM compared to NGT (Fig. 2A). Since PMA/ionomycin induced non-specific mitogen activation of CD4 + and CD8 + cells, we further evaluated the peripheral CD8 + cells activated by cytomegalovirus (CMV) peptide. Peripheral CD8 + T cells from NGT (n = 5) and DM (n = 8) individuals possessing HLA-A*24:02 were investigated. The CD8 + PD1 + T cells specific to HLA-A*24:02 CMV pp65 peptide were significantly reduced in DM compared with NGT (Fig. 2B). Furthermore, the populations of CD8 + PD1 + T cells producing IFNγ and IFNγ + TNFα stimulated with HLA-A*24:02 CMV pp65 peptide were significantly reduced in DM compared with NGT (Fig. 2C). In DM, multifunctionality of peripheral CD8 + T cells was impaired in response to both non-specific and antigen-specific activation.

### Metformin enhanced multifunctionality of CD8 + T cells in patients with T2D

**Figure 3:**
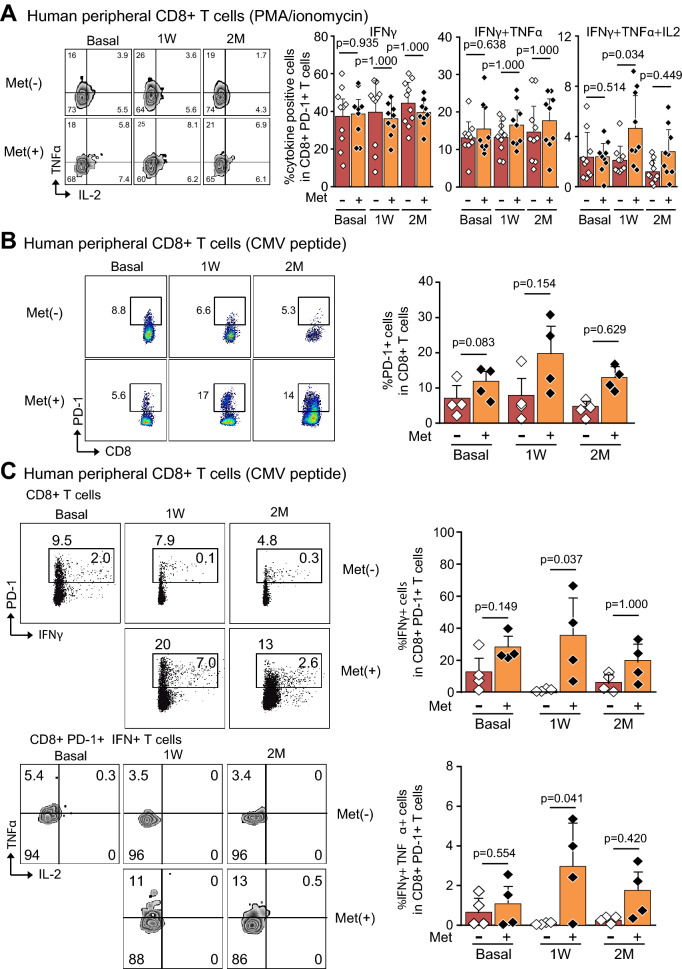
Human peripheral CD8 + T cells isolated from the patients with type 2 diabetes treated with metformin, Met(+), and without metformin, Met(−). (**A**). he percentage of single (IFNγ), double (IFNγ + TNFα), and triple (IFNγ + TNFα + IL2) cytokine producing cells in CD8 + PD-1 + T cells at basal, 1 week (1 W), and 2 months (2 M) after the treatment with and without metformin. Met(−) (n = 10) and Met(+) (n = 9). (**B**) Human peripheral CD8 + T cells stimulated with HLA-A*24:02 CMV pp65 peptide. The percentage of PD-1 + cells in CD8 + T cells in Met(+) and Met(−) is shown. Met(−) (n = 4) and Met(+) (n = 4). (**C**) Human peripheral CD8 + T cells stimulated with HLA-A*24:02 CMV pp65 peptide. The percentage of IFNγ and IFNγ + TNFα producing cells in CD8 + PD-1 + T cells is shown. Met(−) (n = 4) and Met(+) (n = 4). (**A**, **B**, and **C** upper panel, Bonferroni correction; **C** lower panel, Tukey–Kramer).

We further investigate the therapeutic effects of metformin on peripheral CD8 + T cells in the hospitalized patients with T2D without treatments of metformin. They were randomly assigned to metformin group; Met(+) (n = 9), and non-metformin group; Met(−) (n = 10). During hospitalization for 1–2 weeks, life-style modification and medications were started to achieve the therapeutic goals according to the guideline (online supplementary Table S2 and S3). Peripheral CD8 + T cells were obtained at baseline, 1 week and 2 months after the randomization and initiation of metformin. In gated CD8 + T cells, there were no differences in the populations of IFNγ, IFNγ + TNFα and IFNγ + TNFα + IL2 producing cells in Met(+) and Met(−) (online supplementary Figure S3). However, in gated CD8 + PD1 + T cells, populations of IFNγ + TNFα + IL2 producing cells demonstrated significant increase at 1 week and non-significant increase at 2 months in out-patient clinic (Fig. 3A), suggesting the importance of both metformin administration and life style modification to maintain the multiple functionality of CD8 + cells. Next, we investigated the CD8 + PD1 + T cells specific to HLA-A*24:02 CMV pp65 peptide and they increased at 1 week and 2 months after metformin treatment, although they did not reach statistically significant differences (Fig. 3B). Similarly, by the stimulation of HLA-A*24:02 CMV pp65 peptide, CD8 + PD1 + T cells demonstrated significantly higher population of IFNγ + and IFNγ + TNFα producing cells in Met(+) group, and they were partially maintained at 2 months with no statistical significance (Fig. 3C).

The phosphoenolpyruvate sustains anti-tumor effector function of T cells by repressing sarco/ER Ca^2+^-ATPase (SARCA) activity and increasing cytokine production. In addition, glyceraldehyde 3-phosphate and glyceraldehyde 3-phosphate dehydrogenase (GAPDH) have been implicated in the regulation of IFNγ translation. Since glycolytic metabolites are involved in the cytokine production in T cells, we next investigated the glycolysis and oxidative phosphorylation by Flux Analyzer using mouse and human samples.

**Figure 4:**
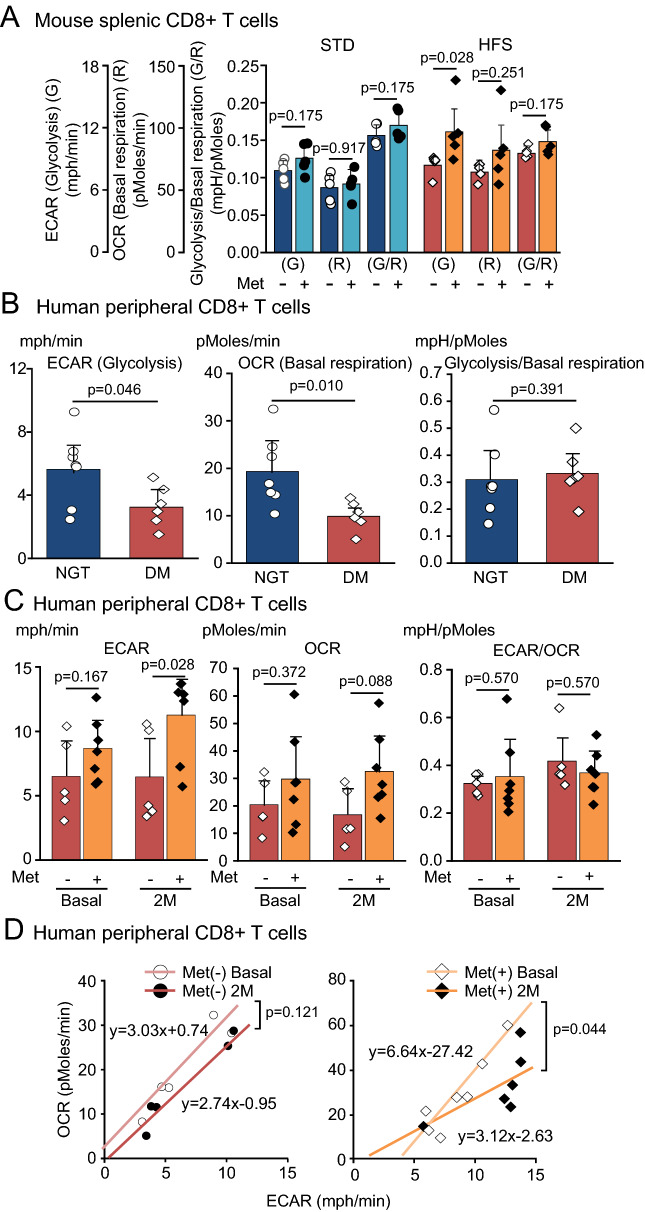
Extracellular acidification rate (ECAR) (glycolysis) and oxygen consumption rate (OCR) (basal respiration) demonstrated by Flux Analyzer using mouse and human CD8 + T cells. (**A**) ECAR (glycolysis) and OCR (basal respiration) in mouse splenic CD8 + T cells derived from C57BL/6JJcl fed with STD and HFS treated with and without metformin. STD (n = 5), HFS (n = 5), STD + Met (n = 5), and HFS + Met (n = 5). (**B**) ECAR (glycolysis) and OCR (basal respiration) in NGT (n = 7) and DM (n = 6) in human peripheral CD8 + T cells. (**C**,**D**) ECAR and OCR at basal and 2 months after the treatment with and without metformin. Human peripheral CD8 + T cells were subjected to Flux Analyzer in the presence of glucose in culture media. Met(−) (n = 5) and Met(+) (n = 7). (**A**–**C**, Mann–Whitney U test; **D**, Covariance analysis).

The 4-week-old C57BL/6JJcl mice fed with STD and HFS for 12 weeks. They were further free access to water with and without metformin for 2 weeks. Finally, CD8 + splenic T cells were isolated from 18-week-old C57BL/6JJcl mice. Under HFS, glycolysis was significantly increased, while basal respiration was not altered by the treatment with metformin (Fig. 4A). Under STD, glycolysis, basal respiration and glycolysis/basal respiration ratio were not altered by the treatment with metformin (Fig. 4A). By the comparison between STD and HFS groups without the treatment of metformin, glycolysis and basal respiration were not altered, while glycolysis/basal respiration ratio was significantly reduced in HFS group (online supplementary Figure S4).

Human peripheral CD8 + T cells were isolated from NGT (n = 7) and DM (n = 6) without treatment of metformin. Both glycolysis and basal respiration were reduced in DM compared with NGT (Fig. 4B and online supplementary Figure S5). The peripheral CD8 + T cells in DM demonstrated reduction in glycolysis and also in mitochondrial function and it may reflect the poor glycemic control with HbA1c levels of 9%. Peripheral CD8 + T cells were obtained at baseline and 2 months after the randomization, subjected to Flux Analyzer, and ECAR/OCR was measured in the presence of glucose in the culture media. ECAR was significantly increased 2-month treatment with metformin, while OCR and ECAR/OCR ratio was not altered (Fig. 4C). In Met(−) group, the slope of x-axis (ECAR) and y-axis (OCR) was not altered, while it was significantly tilted downward after the 2 months treatment in Met(+) group (Fig. 4D).

## Discussion

In the current investigation, we demonstrated that circulating CD8 + PD-1 + population derived from the patients with T2D was reduced. Circulating CD8 + T cells were characterized by reduced glycolysis and decreased basal respiration of mitochondria, and CD8 + PD-1 + T cells demonstrated impaired multiple cytokine production. We newly identified that the impairment of the metabolism-immune function axis of CD8 + PD-1 + T cells in T2D leads to the reduction of MHC-restricted antigen-specific response and cytokine production. The experiments in HFS-fed mice demonstrated reduced glycolysis/basal respiration ratio and impaired multifunctionality of CD8 + PD-1 + T cells in spleen and tumor and it suggested that development and progression of neoplasm is promoted by impairment of metabolism-immune function axis of CD8 + PD-1 + T cells. Although PD-1 is well-known as a key molecule for T cell exhaustion during cancer and chronic infection, it is also a marker for antigen-stimulated activation of both T and B cells^19^. PD-1 is expressed during the early phase and T cell activation when naïve CD8 T cells differentiate into effector cells producing high levels of cytokines IFNγ and TNFα upon stimulation^20^. The dynamic PD-1 expression during the acute and chronic antigen stimuli is mediated by transcription factors, DNA methylation, and histone modification in CD8 + T cells^21^. The reduction of CD8 + PD-1 + cells in the patients with T2D suggested the lower avidity contributes the reduction of cytokine production. We also clearly demonstrated the link between the reduced glycolysis and impaired cytokine production, *i.e.* lowered avidity of CD8 + PD-1 + cells in T2D. Transcription factor, interferon regulatory factor 4 (IRF4), was induced depending on affinity for the TCR and up-regulated the expressions of key enzymes required for the aerobic glycolysis during the clonal expansion and activation^22^. Very low affinity TCR ligands are sufficient to induce the rapid clonal expansion, but the expansion period and burst size tightly correlates with the strength of TCR ligation, which is required for the sustained expansion of CD8 + T cells^23^. Effector CD8 T cells maintain a highly glycolytic metabolism, while memory CD8 T cells rely on fatty acid oxidation and mitochondrial oxidation^24^. The downregulated expression of PD-1 has been also reported in circulating CD4 + T cells, CD8 + T cells, natural killer cells, and monocytes in the patients with T2D^25^. Thus, we concluded that CD8 T cells in the patients with diabetes cannot maintain elevated levels of glycolysis and high avidity upon TCR stimulation. This process may be mediated by the alterations in uptake and catabolism of glucose generate substrates such as acetyl-CoA, and activity modulators such as α-ketoglutarate, succinate, fumarate, and lactate used by choromatin modifying enzymes^26^.

The dysfunction of CD8 + T cells, *i.e.* impaired multiple cytokine production, in diabetes were confirmed by the stimulation with the combination of phorbol 12-myristate 13-acetate (PMA) and ionomycin. PMA is protein kinase C activator, while ionomycin is highly selective Ca^2+^ ionophore and it increases intracellular Ca^2+^ concentrations by the transport across biological membranes. The combination of PMA and ionomycin activates the transcription factors NF-kB and NFAT leading to the production of the cytokines, such as IL2. Under non-specific stimulation independent of TCR-mediated pathway, we demonstrated simultaneous reduction of glycolysis and cytokine production in CD8 + PD-1 + cells derived from patients with T2D. Glycolytic metabolite phosphoenolpyruvate sustains Ca^2+^-NFAT signaling and stimulates the cytokine production by repressing sarco/ER Ca^2+^-ATPase (SERCA) activity^27^. Furthermore, the glycolytic enzyme GAPDH binds to AU-rich elements within the 3′ UTR of IFNγ mRNA and reduces protein translation if GAPDH is not occupied glycolytic metabolic pathway^28^. The reduced glycolytic activities of CD8 + PD-1 + cells derived from patients with T2D contribute the reduction of cytokine production. To evaluate the TCR specific signal transduction, the previously defined QYDPVAALF peptide epitope from pp65, the only epitope recognized by every HLA A24:02-positive donor^29^, was used to stimulate CD8 + PD-1 + cells derived from HLA A24:02-positive patients withT2D. Similar to the stimulation with PMA/ionomycin, multiple cytokine production was prominently abolished in the patients. In addition to susceptibility to viral infection, we also demonstrated reduced cytokine production in tumor infiltrating CD8 + cells in mouse diet-induced model of obesity. Intriguingly, cytokine production of splenic CD8 + cells rather increased in this model. Immune exhaustion is caused by tumor cell-expressing immune check point receptor ligands such as PD-L1 and galectin-9 by binding to PD-1 and Tim-3 on tumor infiltrating lymphocytes, respectively^30^. The reduction of glucose, upper glycolytic intermediates, and amino acids in tumor tissues^31^ and persistent antigen exposure^32^ cause the immune exhaustion^33,34^, where mTOR activity, glycolytic capacity, and IFN-γ production are dampened^8^. The results suggested that the metabolic changes and impaired cytokine production in CD8 + T cells is involved in susceptibility to virus infection and development of malignancies in diabetes.

Metformin is known to inhibit complex I in mitochondria, oxidative phosphorylation, and ATP production, which is compensated by increasing glycolysis in cancer cells^35^. However, the energy status and cytokine production of immune cells in diabetes were not well-explored and we newly identified the insufficient glycolysis and impaired multiple cytokine production in circulating CD8 + PD-1 + cells. We further administered metformin to the patients and demonstrated that the energy reprogramming shifting to aerobic glycolysis by metformin recovers the avidity of CD8 + PD-1 + cells in response to both TCR-mediated specific and PMA/ionomycin mediated non-specific stimuli. We also administered metformin to the melanoma bearing mice under HFS and STD chow. Although basal mitochondrial respiration was not altered by metformin, glycolysis was significantly enhanced in splenic CD8 + T cells derived from metformin-treated mice under HFS chow, which resulted in the recovery of cytokine producing CD8 + T cells. Naïve and memory T cells mainly depend on the mitochondrial oxidative phosphorylation for survival and migration^36^, while effector T cells utilize glycolysis converting glucose to lactate to drive robust cell proliferation^37^. The line of evidence suggests that the energy reprogramming by metformin directly link to the recovery of multiple cytokine production of CD8 + T cells.

There are some limitations in current investigation. Firstly, NGT and DM were matched for age and gender, DM demonstrated higher body mass index, total cholesterol, and low density lipoprotein cholesterol. Therefore, we are not able to completely exclude the influence of obesity and dyslipidemia on cytokine production and glycolysis of CD8 + T cells. Secondly, HbA1c was lower in Met(+) at the final observation and the lowered blood glucose levels may reverse cytokine production and glycolysis besides the direct action of metformin. Thirdly, the low-fat and nutrient matched chow (D12329) is recommended for the control for HFS chow (D12331); however, STD (MF chow) was used in the current study and it may not be an appropriate control.

Taken together, circulating CD8 + T cells in the patients with T2D demonstrated insufficient glycolysis, decreased basal respiration, and impaired multiple cytokine production. Furthermore, the administration of metformin recovers the antigen-specific signaling and cytokine production by increasing the aerobic glycolysis activity. Although hyperglycemia, insulin resistance and obesity have been speculated to be involved in cancer development^3^, the current investigation specifically demonstrates that dysfunction of CD8 + T cells contributes the susceptibility to cancers and infections in the patients with T2D. Furthermore, we shed the lights into the mechanism for epidemiological evidence of cancer prevention by metformin^11^, where the shift to glycolysis and recovered CD8 + T cell avidity in response to antigen-TCR specific stimuli were presented in the current investigation. In previous report, OCR and ECAR were investigated in PBMCs derived from the patients with type 2 diabetes^38^, we firstly demonstrated T cell subset-specific functional linkage between glucose metabolism and cytokine production. In next stage, the dual approach of immunometabolism for various immune mediated cells such as CD4 T cells and macrophages further promotes development of therapeutics in diabetes and its complications.

## Methods

### Animals

C57BL/6JJcl mice (CLEA Japan, Tokyo, Japan) and B6.CB17-Prkdc^scid^/SzJ (SCID) mice (The Jackson Laboratory, Bar Harbor, ME) were maintained with free access to chow and water. The mice were fed standard chow diet (STD) (MF, Oriental Yeast, Japan) or high fat-high sucrose diet (HFS: Protein: 16.4% kcal, Fat: 58% kcal, Carbohydrate: 25.5% kcal, Energy Density: 5.56 kcal/g) (D12331, Research Diets, New Brunswick, NJ).

For tumor growth assay, 4-week-old C57BL/6JJcl and B6.CB17-Prkdc^scid^/SzJ male mice were fed with STD or HFS for 6 weeks and injected with 4 × 10^5^ B6 OVA-gene introduced B16 melanoma MO5 cells. Seven days after injection of MO5 cells, they were divided into 4 groups fed with (1) STD with water (STD), (2) STD with 5 mg/dl metformin (WAKO, Tokyo, Japan) water (STD + Met), (3) HFS with water (HFS), and (4) HFS with 5 mg/dl metformin water (HFS + Met) for 4 weeks. Mice that received metformin at 5 mg/ml achieved 1.70 μg/mL, which was consistent with plasma concentrations in patients (0.5–2 μg/mL)^39^. Tumor infiltrating lymphocytes (TILs) were isolated at 48 h after the administration of metformin and subjected to cytokine assay.

4-week-old C57BL/6JJcl mice were fed with STD and HFS for 12 weeks and they were divided into STD, STD + Met, HFS, and HFS + Met for 2 weeks. Then, splenocytes were isolated and subjected to cytokine assay and Extracellular Flux Analyzer.

### Tumor growth assay

The mice were intradermally inoculated with 4 × 10^5^ B6 OVA-gene introduced B16 melanoma MO5 cells in 100 μl of RPMI on the back with a 27-gauge needle after the hair shaved. The tumor diameter was measured with Vernier calipers in every 3 days until 25 days after the injection, and the volume of the tumor was calculated using a formula V = W^2^ × L)/2 (V = volume, W = Minor axis, L = Major axis).

### Oral glucose tolerance test (OGTT)

The 4 groups of 16-week-old C57BL/6JJcl mice under STD, STD + Met, HFS, and HFS + Met for 12 weeks were fasted for 16 h and subjected to Oral Glucose Tolerance Test (OGTT). A glucose (2 g/kg) solution was given and blood samples were taken from tail veins at basal, 30, 60, and 120 min after the administration of the glucose solution.

### Cytokine assay

Isolated mouse splenocytes without red blood cells or tumor infiltrating lymphocytes (TILs) were stimulated in the presence of 50 ng/ml Phorbol 12-Myristate 13-acetate (PMA) (Sigma-Aldrich, St. Louis, MO) and 2 µM Ionomycin (Sigma-Aldrich), and treated with BD Golgi Stop (BD Biosciences, San Jose, CA) for 6 h at 37 °C in a humidified atmosphere of 5% CO_2_^13,40^. After the culture, the cells were stained with fluorescent dye and monoclonal antibodies (mAbs): Zombie Aqua, CD3 (BV521), and CD8 (APC/Fire) mAbs (BioLegend, San Diego, CA). Next, the cells were resuspended in BD Cytofix/Cytoperm solution (BD Biosciences). The cells were further stained in BD Perm/Wash buffer with IFN-γ (FITC), TNF-α (BV421), and IL-2 (PE/Cy7) mAbs. Finally, these cells were analyzed by FACS Canto II flow cytometer (BD Biosciences).

Human peripheral blood mononuclear cells (PBMCs) were stimulated in the presence of 50 ng/ml PMA and 2 µM Ionomycin, and then treated with BD Golgi Stop for 6 h at 37 °C in a humidified atmosphere of 5% CO_2_. After the culture, the cells were stained with fluorescent dye and monoclonal antibodies (mAbs): Zombie Aqua (BV521), CD8 (APC/Fire), PD-1 (PE/Cy7) mAbs (BioLegend, San Diego, CA). Next, the cells were resuspended in BD Cytofix/Cytoperm solution (BD Biosciences). The cells were further stained in BD Perm/Wash buffer with IFN-γ (FITC), TNF-α (BV421), and IL-2 (APC) mAbs. Finally, these cells were analyzed by FACS Canto II flow cytometer (BD Biosciences).

### Extracellular acidification rate (ECAR) and oxygen consumption rate (OCR)

CD8 + T cells were purified from mouse splenocytes as well as human peripheral blood mononuclear cells (PBMCs) from the patients with T2D (DM) and subjects with normal glucose tolerance (NGT) using MACS (Magnetic-activated cell sorting). The CD8 + T cells were suspended in XF medium (non-buffered RPMI 1640 containing 10 mM glucose, 2 mM L-glutamine, and 1 mM sodium pyruvate) and 7 × 10^5^ cells seeded in each well. Then, ECAR and OCR were measured with XFe96 Extracellular Flux Analyzer (Agilent Technologies, Santa Clara, CA) by serial injections of following regents at final concentrations of 1 μM oligomycin, 8 μM carbonyl cyanide-*p*-trifluoromethoxyphenylhydrazone (FCCP), 1 μM Rotenone/Antimycin, and 100 mM 2-deoxy-D-glucose (2-DG).

In clinical randomized control trial, CD8 + T cells were isolated from human PBMCs of the patients with T2DM treated with metformin and without metformin in the following clinical study. The CD8 + T cells were suspended in XF medium (non-buffered RPMI 1,640 containing 10 mM glucose, 2 mM L-glutamine, and 1 mM sodium pyruvate) and 7 × 10^5^ cells seeded in each well. Then, ECAR and OCR were measured with XFe96 Extracellular Flux Analyzer.

### Metformin and immune exhaustion in type 2 diabetes; randomized control trial (METRO)

To investigate whether the production of multiple cytokines from CD8 + cells is impaired under high glucose conditions in the patients with T2D and whether metformin improves the functional abnormalities of CD8 + cells, clinical trial “Metformin and immune exhaustion in type 2 diabetes; Randomized Control Trial (METRO)” was performed (UMIN000018637). METRO study was approved by Okayama University Graduate School of Medicine, Dentistry and Pharmaceutical Sciences and Okayama University Hospital, Ethics Committee (m05004). We recruited the hospital in-patients with T2D, (1) who understood the purpose, methods and side effects of metformin and gave written informed consents, (2) who were able to claim the unwanted symptoms and signs, (3) who were not less than 20 years old and not more than 75 years old, and (4) who did not receive metformin. The patients with lactic acidosis, moderate renal dysfunction, hemodialysis, severe liver dysfunction, shock, heart failure, cardiac infarction pulmonary embolism, respiratory failure, hypoxia, alcohol intake, dehydration, diarrhea with dehydration, vomiting, severe ketosis, diabetic coma and precoma, type 1 diabetes, severe infection, pre- and post-operation, severe trauma, malnutrition, famine, panhypopituitarism, adrenal failure, pregnancy, and allergic to biguanides were excluded.

We randomly assigned the patients with T2D to metformin group, Met(+) (n = 9), non-metformin group, Met(−) (n = 10) (online supplementary Figure S6). In the metformin group, the initial dose was 500 mg/day (250 mg twice daily), and the dose was increased up to 1,500 mg/day (500 mg thrice daily) over 2 weeks by confirming the absence of adverse effects. Any antidiabetic drugs other than metformin were allowed to be used in both groups to achieve the blood glucose control. Blood samples were collected at baseline, 1 week at hospital and 2 months at out-patient clinic, to evaluate the function of CD8 + T cells. Blood samples from subjects with normal glucose tolerance (n = 15) were used as control.

### Separation and storage of blood cells

About 20 ml blood samples were collected from the patients with T2D and the subjects with normal glucose tolerance. PBMCs were isolated by density gradient centrifugation using a Ficoll-Paque (GE Healthcare Bio-Sciences, Piscataway, NJ). The 2 × 10^6^ PBMCs were suspended in 500 µl of BAMBANKER (LYMPHOTEC, Tokyo, Japan) media, and the cells are placed in cryotubes for freezing and preservation. Then, the cells are frozen and preserved at -180 °C for further use.

### Cytomegalovirus (CMV) assay

Human PBMCs were stimulated with 1 μg/ml HLA-A*24:02 CMV pp65 peptide in AIM-V media (Thermo Fisher Scientific, Waltham, MA) supplemented with 5% pooled serum and 10 U/ml IL-2) on 24-well plates and incubated for 2 weeks at 37 °C in a humidified atmosphere of 5% CO_2_. Then, the half volume of medium was changed with freshly prepared AIM-V media containing 5% pooled serum and 10 U/ml IL-2 in every 2 days and cultured for additional 1 week (cultured PBMCs). After the removal of CD8 + T cells by CD8 MicroBeads (MiltenyiBiotec), 1.0 × 10^6^ PBMCs were incubated in AIM-V containing 5% pool serum and IL-2 10U/ml in the presence (CMV +) and absence (CMV−) of 1 μg/ml HLA-A*24:02 CMV pp65 peptide for 1 h. The CMV + and CMV− were mixed with equal cell numbers of “cultured PBMCs” and treated with BD Golgi Stop for 6 h, respectively. After the culture, the mixed cells were stained with Zombie (BV521), CD8 (APC/Fire), PD-1 (PE/Cy7), Tim-3 (PE) mAbs. Next, they were resuspended in BD Cytofix/Cytoperm solution. There after cells were stained in BD Perm/Wash solution with IFN-γ (FITC), TNF-α (BV421), IL-2 (APC) mAbs and analyzed by FACS Canto II flow cytometer (BD Biosciences).

### Statistical analysis

All results are expressed as means ± standard deviation (SD). Computations assumed that all groups were samples from populations with the same scatter. The significance was determined by Mann–Whitney U test and two-way repeated measure ANOVA with Bonferroni method or Tukey–Kramer, and analysis of covariance (ancova) for comparison of 2 regression lines using SPSS software. A *P* value of < 0.05 was considered significant.

### Ethical approval

The animal experiments were approved by the Animal Care and Use Committee of the Department of Animal Resources, Advanced Science Research Center, Okayama University (OKU-2016295, OKU-2017310, and OKU-2019350). All animal experiments were performed in accordance with relevant guideline and regulations.

For human research, METRO study was approved by Okayama University Graduate School of Medicine, Dentistry and Pharmaceutical Sciences and Okayama University Hospital, Ethics Committee (m05004) and performed in accordance with relevant guidelines/regulations.

### Informed consent

The informed consent was obtained from all participants.

## Supplementary information

Supplementary Information.
