# Supplementary material for: Dysfunction of CD8 + PD-1 + T cells in type 2 diabetes caused by the impairment of metabolism-immune axis
Source: Sci Rep. 2020 Sep 10;10:14928. doi: 10.1038/s41598-020-71946-3 (PMC7484782; doi:10.1038/s41598-020-71946-3)
Supplement: Supplementary file 1 — Supplementary Information. [file 41598_2020_71946_MOESM1_ESM.pdf]

Supplementary Data

Dysfunction of CD8+PD-1+ T cells in type 2 diabetes caused by the impairment of metabolism-immune axis

Ichiro Nojima [1], M.D., Shingo Eikawa[2 8], Ph.D., Nahoko Tomonobu[3], M.S., Yoshiko Hata[1], M.D., Nobuo Kajitani[4], M.D., Ph.D., Sanae Teshigawara[5], M.D., Ph.D., Satoshi Miyamoto[6], M.D., Ph.D., Atsuhito Tone[5], M.D., Ph.D., Haruhito A. Uchida[7], M.D., Ph.D., Atsuko Nakatsuka[1], M.D., Ph.D., Jun Eguchi[1], M.D., Ph.D., Kenichi Shikata[6], M.D., Ph.D., Heiichiro Udono[8], M.D., Ph.D., and Jun Wada[1], M.D., Ph.D.

Supplementary Table 1 Clinical characteristics of the patients with type 2 diabetes (DM) and subjects with normal glucose tolerance (NGT)

	Group	Median (95th percentile)	p value*
Age (year)	NGT	50.9(44-60)	0.452
	DM	54.8(49-60)	
Body mass index (kg/m^2)	NGT	23.4(22.4-24.3)	0.023
	DM	26.4(24.3-28.5)	
Hemoglobin A1c (%)	NGT	6.0(5.8-6.1)	<0.001
	DM	9.7(8.9-10.6)	
Fasting plasma glucose (mmol/L)	NGT	5.07(4.78-5.35)	<0.001
	DM	9.02(7.62-10.43)	
C-peptide (nmol/L)	NGT	0.52(0.36-0.68)	0.790
	DM	0.60(0.39-0.80)	
Total cholesterol (mmol/L)	NGT	4.41(3.93-4.90)	0.005
	DM	5.45(4.96-5.93)	
Low density lipoprotein cholesterol (mmol/L)	NGT	2.57(2.21-2.89)	0.004
	DM	3.41(3.04-3.78)	
High density lipoprotein cholesterol (mmol/L)	NGT	1.34(1.20-1.48)	0.368
	DM	1.33(1.11-1.54)	
Triglyceride (mmol/L)	NGT	1.13(0.80-1.47)	0.30
	DM	1.70(1.24-2.17)	
Aspartate aminotransferase (IU/l)	NGT	19.8(15.7-23.9)	0.141
	DM	25.9(19.0-32.8)	
Alanine aminotransferase (IU/l)	NGT	22.7(16.0-29.4)	0.205
	DM	30.2(19.7-40.7)	
Urea nitrogen (mmol/L)	NGT	5.21(4.63-5.81)	0.548
	DM	5.25(4.57-5.93)	
Creatinine (µmol/L)	NGT	61.3(55.4-67.2)	0.548
	DM	85.6(74.2-97.1)	

*Mann-Whitney U test

Supplementary Table 2 Clinical characteristics of the patients with type 2 diabetes in METRO study

	Group	Basal	p value	1 week	p value	2 months	p value*
Age (year)	Met(-)	54.1(47-64)	0.849				
	Met(+)	55.6(45-63)					
Body mass index (kg/m^2)	Met(-)	26.5(23.0-30.0)	0.569	26.1(22.9-29.2)	0.790	25.8(22.0-27.2)	0.849
	Met(+)	26.3(23.5-29.0)		25.4(22.7-28.0)		24.6(22.4-26.8)	
Hemoglobin A1c (%)	Met(-)	10.4(9.2-11.5)	0.074			7.6(7.2-7.9)	0.003
	Met(+)	8.9(7.7-10.1)				6.7(6.2-7.0)	
Fasting plasma glucose (mmol/L)	Met(-)	9.45(7.35-11.54)	0.470	7.46(6.85-8.07)	0.006	8.21(6.55-9.87)	0.624
	Met(+)	8.51(6.24-10.77)		5.9(5.04-6.75)		7.88(5.98-9.78)	
C-peptide (nmol/L)	Met(-)	0.51(0.32-0.71)	0.676			1.08(0.72-1.44)	0.191
	Met(+)	0.71(0.28-1.13)				0.79(0.45-1.14)	
Total cholesterol (mmol/L)	Met(-)	5.25(4.31-6.18)	0.342	4.61(3.77-5.46)	0.063	4.27(3.77-4.76)	0.022
	Met(+)	5.61(5.01-6.20)		5.49(4.82-6.15)		5.47(4.63-6.31)	
Low density lipoprotein cholesterol (mmol/L)	Met(-)	3.64(3.13-4.15)	0.160	3.57(3.04-4.11)	0.068	3.44(2.73-4.15)	0.005
	Met(+)	3.13(2.53-3.73)		2.74(2.19-3.30)		2.38(2.11-2.65)	
High density lipoprotein cholesterol (mmol/L)	Met(-)	1.28(1.03-1.54)	0.790	1.26(1.06-1.46)	0.909	1.39(1.09-1.68)	0.595
	Met(+)	1.38(0.96-1.81)		1.33(0.89-1.76)		1.36(0.95-1.78)	
Triglyceride (mmol/L)	Met(-)	1.68(1.04-2.33)	0.849	1.40(0.88-1.91)	0.223	2.04(1.17-2.90)	0.369
	Met(+)	1.73(0.90-2.56)		1.17(0.66-1.68)		1.61(0.64-2.57)	
Aspartate aminotransferase (IU/l)	Met(-)	26.6(20.6-32.7)	0.094	26.8(20.2-33.4)	0.147	20.9(16.4-25.4)	0.325
	Met(+)	25.0(9.3-40.7)		25.1(9.9-40.3)		19.4(12.7-26.1)	
Aanine aminotransferase (IU/l)	Met(-)	31.2(22.3-39.1)	0.073	34.1(22.4-43.7)	0.119	25.0(19.5-30.6)	0.079
	Met(+)	29.0(4.47-53.5)		34.6(1.46-67.7)		23.4(5.97-40.9)	
Urea nitrogen (mmol/L)	Met(-)	4.82(4.03-5.61)	0.342	4.69(3.90-5.47)	0.160	5.20(4.05-6.34)	0.513
	Met(+)	5.77(4.49-7.05)		5.59(4.34-6.85)		5.42(4.50-6.34)	
Creatinine (µmol/L)	Met(-)	64.0(52.8-75.2)	0.909	71.7(58.1-85.3)	0.621	71.2(58.4-83.9)	0.838
	Met(+)	65.3(48.7-81.9)		77.5(58.4-96.6)		70.7(55.1-86.3)	
Lactic acid (mmol/L)	Met(-)	1.35(0.73-1.98)	0.849	1.38(0.30-2.46)	0.909	1.24(0.92-1.55)	0.624
	Met(+)	1.58(0.26-2.91)		1.15(0.65-1.65)		1.41(0.85-1.97)	
Pyruvate (µmol/L)	Met(-)	146(94-198)	0.939	97(72-122)	0.237	130(67-194)	0.462
	Met(+)	158(73-243)		120(82-158)		181(107-254)	

Metformin group; Met(+) (n=9), and non-metformin group; Met(-) (n=10).

***Mann-Whitney U test

Supplementary Table 3 Medications in the patients with type 2 diabetes in METRO study

Drug Class and Name	Metformin group; Met(+) (n=9)			Non-metformin group; Met(-) (n=10)		
	Basal	1 week	2 months	Basal	1 week	2 months
Metformin	0	9*	9†	0	0*	0†
Sulfonylureas	3	2	1	1	0	0
Thiazolidinediones	1	0	0	0	0	0
α-Glucosidase inhibitor	3‡	4	3	0‡	1	1
Dipeptidyl peptidase-4 inhibitor	3	3	4	6	4	4
Glucagon like peptide-1 receptor agonist	0	1	1	1	3	4
Sodium-dependent glucose transporter 2 inhibitor	1	3	2	0	3	5
Insulin	4	4	3	7	8	3
Life-style modification only	2	0	0	1	0	0

*, $p<0.001$; †, $p<0.001$; ‡, $p=0.047$. Met(+) *vs* Met(-).

Supplementary Figure S1

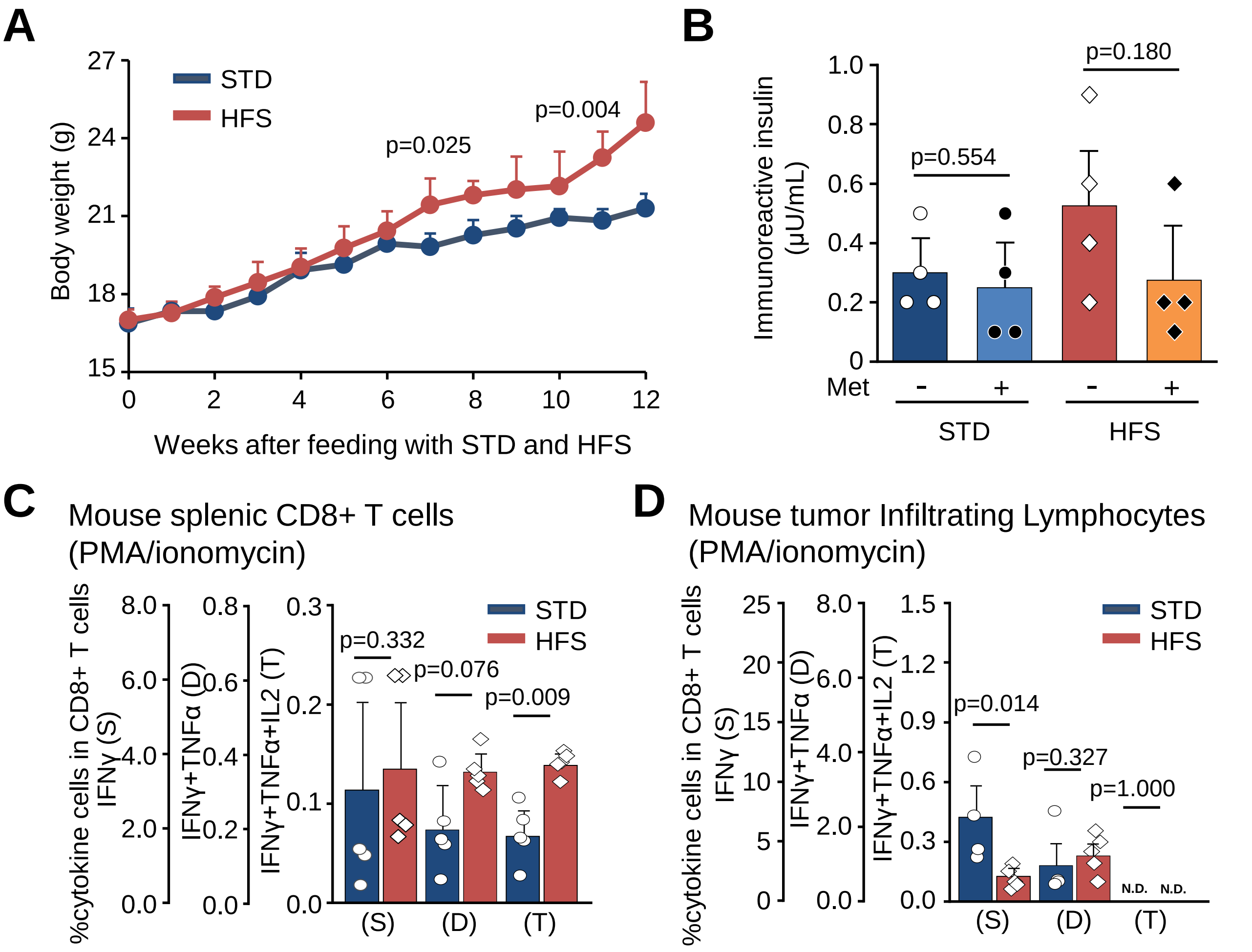

Supplementary Figure S1 16-week old C57BL/6JJcl mice fed with standard chow (STD; n= 6), high fat-high sucrose chow (HFS; n=6). **A**. Time course of body weight. STD (n=6) and HFS (n=6). **B**. Immunoreactive insulin 12 weeks after the feeding with STD and HSF. STD (n=5), HFS (n=5), STD + Met (n=4), and HFS + Met (n=5). **C.** Mouse splenic CD8+ T cells treated with PMA/ionomycin. The percentage of single (S) IFNγ, double (D) IFNγ+TNFα, and triple (T) IFNγ+TNFα+IL2 cytokine producing cells in CD8+ T cells. STD (n=5) and HFS (n=5). **D**. Mouse tumor infiltrating lymphocytes (CD8+ T cells) treated with PMA/ionomycin. The percentage of single IFNγ (S), double (D) IFNγ+TNFα, and triple (T) IFNγ+TNFα+IL2 cytokine producing CD8+ T cells. STD (n=5) and HFS (n=5). (**A**-**D**, Mann-Whitney U test)

Supplementary Figure S2

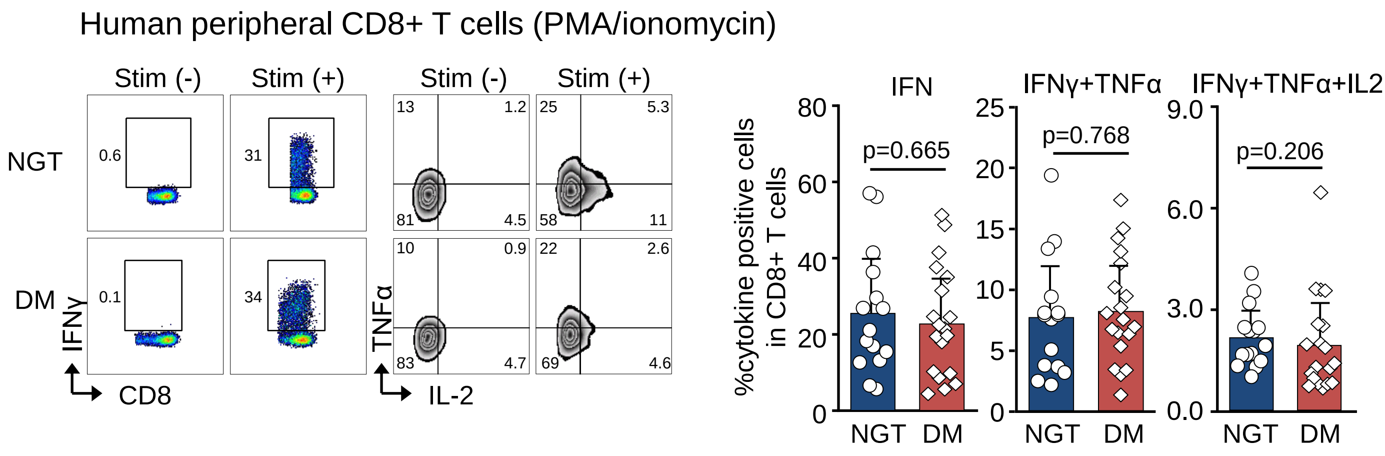

Supplementary Figure S2 The percentage of single IFNγ, double IFNγ+TNFα, and triple IFNγ+TNFα+IL2 cytokine producing cells in CD8+ T cells stimulated with PMA/ionomycin. The CD8+ T cells isolated from the subjects with normal glucose tolerance (NGT) and the patients with type 2 diabetes (DM). NGT (n=15) and DM (n=19). (Mann-Whitney U test)

Supplementary Figure S3

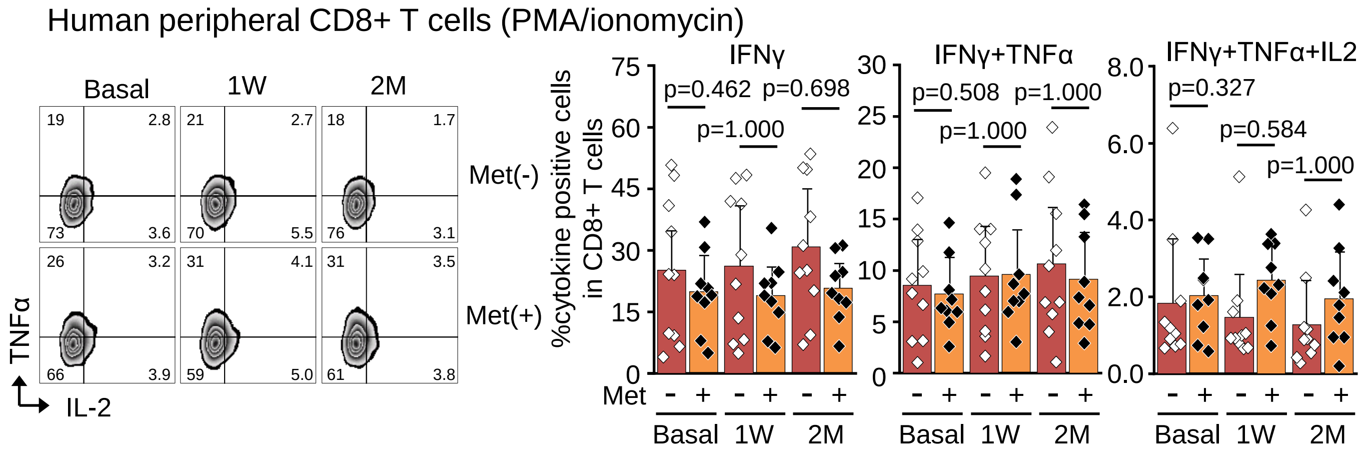

Supplementary Figure S3 The percentage of single (S) IFNγ, double (D) IFNγ+TNFα, and triple (T) IFNγ+TNFα+IL2 cytokine producing cells in CD8+ T cells at basal, 1 week (1W), and 2 months (2M) after the treatment with and without metformin. Human peripheral CD8+ T cells isolated from the patients with type 2 diabetes treated with metformin, Met(+), and without metformin, Met(-) were stimulated with PMA/ionomycin. Met(-) (n=10) and Met(+) (n=9). (Basal, Mann-Whitney U test; 1W and 2M, Bonferroni correction)

Supplementary Figure S4

Mouse splenic CD8+ T cells

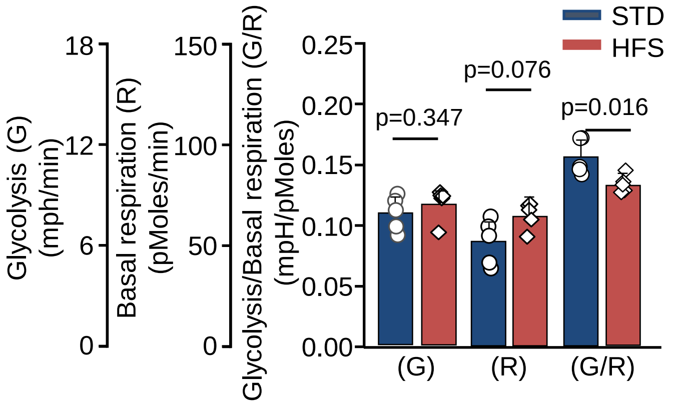

Supplementary Figure S4 Extracellular acidification rate (ECAR) (glycolysis) and oxygen consumption rate (OCR) (basal respiration) demonstrated by Flux Analyzer using mouse CD8+ T cells. ECAR (glycolysis) and OCR (basal respiration) in mouse splenic CD8+ T cells derived from C57BL/6JJcl fed with STD and HFS. STD (n=5) and HFS (n=5). (Mann-Whitney U test)

Supplementary Figure S5

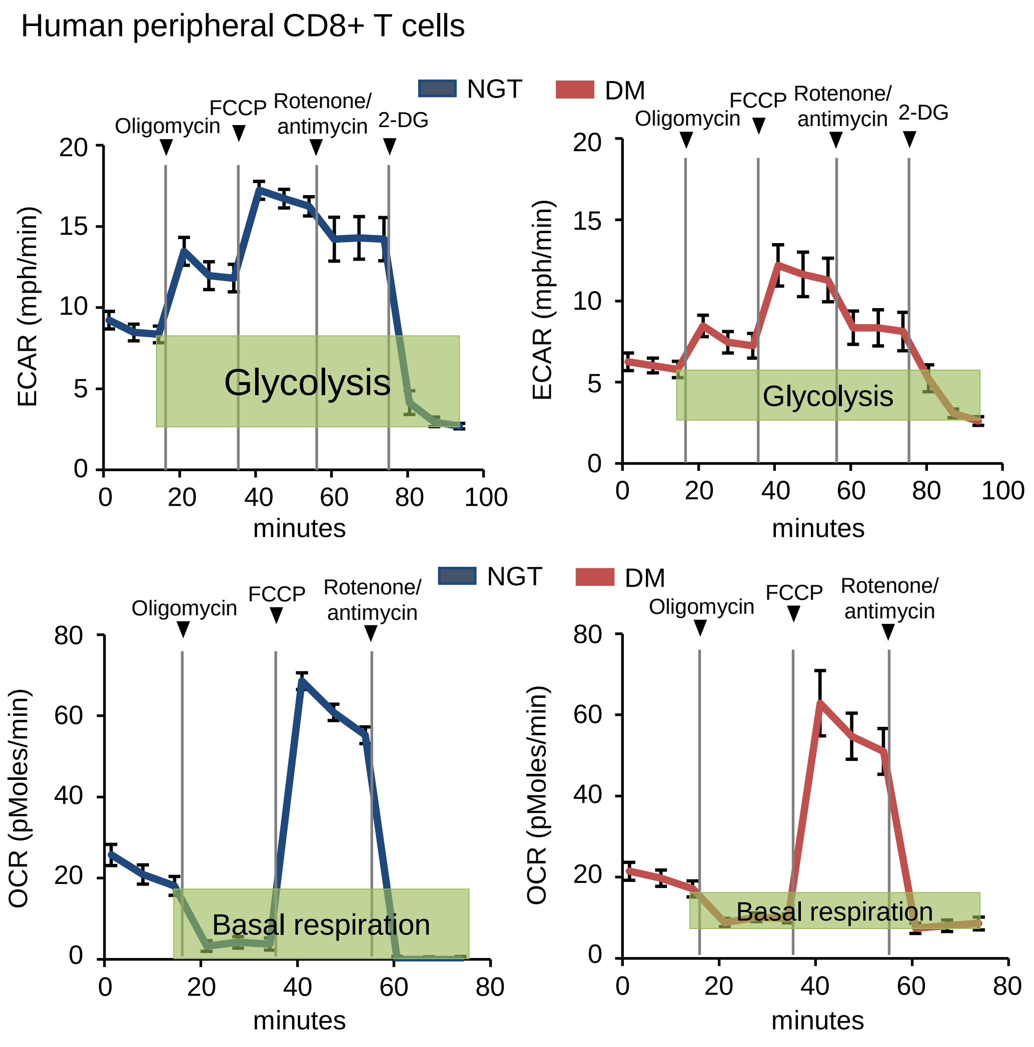

Supplementary Figure S5 Extracellular acidification rate (ECAR) (glycolysis) and oxygen consumption rate (OCR) (basal respiration) demonstrated by Flux Analyzer using human CD8+ T cells. The CD8+ T cells isolated from the subjects with normal glucose tolerance (NGT) and the patients with type 2 diabetes (DM). NGT (n=7) and DM (n=6). FCCP, carbonyl cyanide-*p*-trifluoromethoxyphenylhydrazone; 2-DG, 2-deoxy-D-glucose.

Supplementary Figure S6

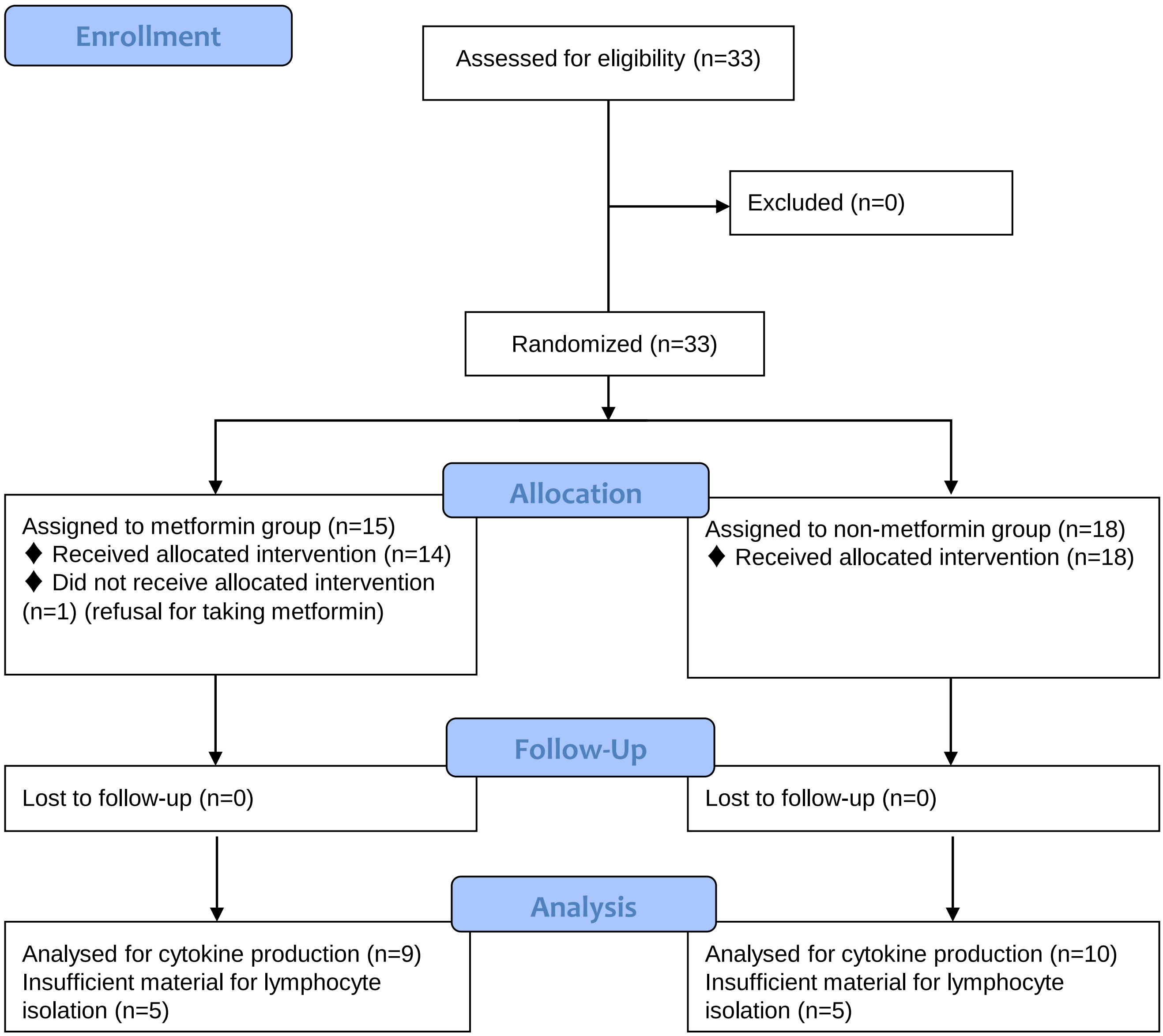

Supplementary Figure S6 CONSORT FLOW Diagram.
